# Validated scoring of halogen bonding in molecular design

**DOI:** 10.1186/1758-2946-6-S1-O17

**Published:** 2014-03-11

**Authors:** MO Zimmermann, A Lange, FM Boeckler

**Affiliations:** 1 Laboratory for Mol. Design & Pharm. Biophysics, Eberhard Karls University Tuebingen, Auf der Morgenstelle 8, 72076 Tuebingen, Germany

## 

As a rather new type of non-covalent interaction, halogen bonding is slowly being integrated into molecular modeling and the drug design process. Aromatic halogenated molecules can theoretically form halogen bonds with any electron donor. Based on quantum chemical calculations, we evaluated the interaction energies between several halobenzenes and the oxygen of *N*-methylacetamide, representing the carbonyl-function of the protein backbone [1,2]. In a ligand-protein complex very rarely optimal interaction geometries are observed. In order to assess all spatial dependencies of the halogen bond with regards to deviations from optimal geometries, our calculations include variations in distance, bond angles and spherical orientation. On this basis we developed a tool for scaffold decoration applicable to any crystal structure or docking result [3]. For every unsubstituted aromatic atom in a ligand the tool determines whether halogenation leads to a favorable halogen bonding interaction with the binding site. A scoring function, derived from a myriad of QM-calculations on the MP2 TZVPP-level of theory, evaluates the quality of the assumed interaction. To assess the prediction accuracy of our scoring function, we initially chose 50 examples from an exhaustive PDB scan randomly, representing the full range of equally distributed halogen bonding scores. For each file, the geometry was then recreated using iodobenzene representing the ligand system and N-methylacetamide as the backbone structure. Thus, the halogen bonding geometry of the database example was transferred as accurately as possible to our small model system and the interaction was calculated as an MP2 single point. The resulting energy was normalized and plotted against the predicted halogen bonding score. With only a few exceptions, most deviations were below 10% leading to an overall r² of 0.87. This highlights that our present scoring function is a blueprint for integration into general empirical scoring functions, which at present ignore halogen bonding interactions. Hence, recognition of halogen bonding will be implemented soon into protein-ligand docking and scoring.

**Figure 1:**
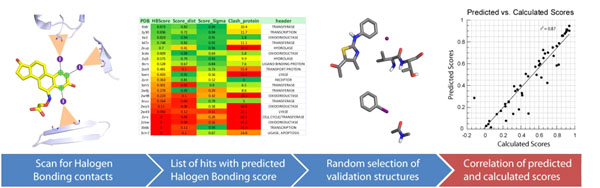


## References

[B1] Wilcken J Comput Aided Mol Des 2012 26 935 945 10.1007/s10822-012-9592-8 22865255

[B2] Wilcken J Med Chem 2013 56 1363 1388 10.1021/jm3012068 23145854

[B3] Zimmermann MO unpublished data

